# Initial Treatment of War Wounds Involving the Knee Joint

**Published:** 1919

**Authors:** 


### *Initial Treatment of War Wounds Involving the Knee Joint.*

By Carleton R. Metcalf, Major M. C.

Dr. Metcalf first takes up the definition of terms used in war surgery, such as : perforating wounds, penetrating wounds, debridement, and sutures, which to surgeons contain the one new term " debridement " or excision of contaminated tissue in a wound track. These are applied in the study of wounds of the knee coming under observation at an evacuation hospital where they were received on an average of fifteen hours after being wounded.

A table is given showing statistics on the type of missile, of wound and of fracture. Shell wounds were more common than bullet wounds, fracture in the joint than non-fracture, penetrating wounds than perforating. Penetrating wounds usually fracture, and fracture of the femur is the most common.

General rules for treatment of all wounds of the knee joint include X-ray examination preceding operation, which latter should be done with a tourniquet after applying an Esmarch bandage from the foot upward. Dressing is applied before removal of tourniquet. A culture should be made from every joint. For joint irrigation, saline solution serves every purpose. The retention of antiseptic fluids in a joint is contra-indicated. Drains should not enter the joint. Cases are best kept under the observation of the operating surgeon for at least ten days after operation.

Non-operative and operative treatment of clean bullet wounds depend on the presence or likelihood of sepsis. Latent sepsis is to be feared if the operation is delayed or the missile is dirty, and shell fragments are more apt to cause it than bullet wounds. Secondarily it depends on existence of fracture, retention of missile, scope of wound and condition of patient. Perforating bullet wounds are usually clean and cause only about 10 o/o of joint infections. Without extensive fragmentation they may be left alone.

Where the wound is bloody, two methods may be adopted: (1) aspirate, make culture, immobilize joint on posterior wire splint, and in a few days active movement may be inaugurated; (2) excise the peri-articular tissues about the entrance and exit of the wounds; allow to drain and at once suture tightly in three layers: synovia, capsule, skin.

Operative treatment of all other types of wound:

(1) Apply Esmarch bandage and tourniquet;

(2) Remove Esmarch bandage;

(3) Prepare operative field;

(4) Debride missile track, both soft tissues and splintered unattached bone. Evacuate fluid and blood-clots;

(5) Remove missile together with bits of foreign material;

(6) Irrigate joint if septic or dirty;

(7) Close synovia, capsule and skin;

(8) Apply dressing of dry gauze or gauze wet with Carrel-Dakin solution, and if necessary a splint;

(9) Remove tourniquet.

In opening a joint it is well when possible to procure complete approach to all injured areas, hence a liberal, lateral incision should be made well outside the patellar edge. If one incision is not sufficient a second is made beyond the opposite patellar edge. Or, one may use a "U" shaped incision, cutting the patellar tendon close to its insertion, though in a somewhat oblique direction. The tendon must always be resutured.

For adequate debridement follow the missile track. When the missile is lodged proximally in condyle or tuberosity, debride the track to the bone, then chisel out bone and missile.

For fracture of the patella avoid resection; dirty bone may be rongeured away. If fragments are large, suture with catgut. In extensive resection do a subperiosteal.

Primary resection is to be avoided. A movable joint seems possible if two-thirds of either or both surfaces remain intact. After a resection immobilize at an angle of 35°.

Primary amputation is demanded for only two conditions:

severe joint infection and complete destruction of blood vessels in the popliteal region.

Closure of operative wounds may be by primary, delayed primary, or secondary suture; or they may be left open for drainage. Primary suture is possible except in presence (*a*) of infection with streptococcus, staphylococcus, gas bacillus, as shown by cultures; (*b*) extensive destruction of capsule; (*c*) large or dirty missiles.

Where primary suture is contra-indicated, the question becomes one of time required to eliminate infection, blood clot or fluid. Delayed primary suture gives as good an end result as primary suture, and the end is gained more speedily. It also eliminates difficulty of diagnosis. The author leans toward delayed primary suture, closing by primary suture only sterile wounds and joint resections.

If sepsis exists very liberal drainage is necessary at either side the patella, and possibly for a considerable time. In the absence of sepsis, a shorter incision at one side of the patella is sufficient.

Initial drainage through the posterior aspect is indicated only when wounds of entrance or exit involve this region, and when tubes are inserted one must bear in mind the possibility of hemorrhage.

Immobilization and mobilization: Closed synovia or extensive destruction of joint connotes temporary mobilization, and an open wound or slight injury requires systematic movement. The Thomas splint affords traction and is therefore best.

Distention of the joint suggests aspiration.

Free use of the joint is indicated in sepsis, as the pus is thus forced out and stiffness prevented.

The author reports results obtained in operating on thirteen cases for various kinds of wounds of the knee.

